# A multi-enzyme producing *Bacillus subtilis* YCXW-01: Isolation, genomic characterization, and potentials in tobacco stem degradation

**DOI:** 10.3389/fmicb.2025.1717865

**Published:** 2025-12-03

**Authors:** Zongcan Yang, Quanbin Zhang, Bo Fu, Tingting Zhang, Yingjie Feng, Sensen Zhao, Huanhuan Wang

**Affiliations:** 1 Technology Center, China Tobacco Henan Industrial Co., Ltd., Zhengzhou, China; 2 College of Tobacco, Henan Agricultural University, Zhengzhou, Henan, China

**Keywords:** *Bacillus subtilis*, cellulase, tobacco stem degradation, genome sequencing, glycoside hydrolase families

## Abstract

This study aimed to isolate a potent cellulase-producing bacterium from tobacco leaves to enhance the utilization value of tobacco stem waste.A strain designated YCXW-01 was isolated using sodium carboxymethyl cellulose as the sole carbon source. It was identified via morphology, physiology, and 16S rRNA sequencing. Growth kinetics, extracellular enzyme activities (cellulase, protease, amylase, xylanase), and whole-genome sequencing were performed. The practical efficacy of its crude enzymes was tested on tobacco stems.The isolate was identified as *Bacillus subtilis*. It exhibited a high cellulase activity of 10.97 U/mL, alongside substantial protease (23.11 U/mL), amylase (28.96 U/mL), and xylanase (23.68 U/mL) production. Genomic analysis revealed genes encoding key glycoside hydrolases (GH1, GH5, GH11, GH43). Treatment of tobacco stems with the crude enzyme extract reduced cellulose content by 35.3%. *B. subtilis* YCXW-01 demonstrates significant potential for tobacco waste biorefining due to its high, multi-enzyme activity and relevant genetic repertoire. The genomic data provide a foundation for elucidating its cellulolytic mechanism and guiding future applications.

## 1 Introduction

Cellulose, a polymer of D-glucose units linked by *β*-1,4-glycosidic bonds, is the most abundant natural polysaccharide and a primary component of plant cell walls (Mengke et al., 2025; Chen et al., 2025). As a major agricultural country, China generates nearly 1.2 billion tons of lignocellulosic biomass. However, a significant portion of this valuable resource is underutilized, often disposed of through burning or landfilling, leading to environmental pollution and resource waste on an annual basis (Farrelly et al., 2025; Pawan et al., 2024). As a method of cellulose utilization, microbial degradation is an environmental protection strategy to solve the problem of resource shortage and environmental pollution (Kant et al., 2024; Kant et al., 2025). Significant progress has been made in the field of screening and characterizing cellulolytic microorganisms (Guodong et al., 2021; Chonglei et al., 2025). For instance, *Bacillus subtilis* C8, isolated from rumen fluid, demonstrated a 43.64% straw degradation rate following mutagenesis (Degao et al., 2024). A thermotolerant microbial consortium has been demonstrated to exhibit a high degree of efficiency in the context of straw composting (Liwen et al., 2025). Genomic analyses have further revealed the diversity of cellulase genes in strains such as *Micrococcus luteus* FJAT-25102 (Jianmei et al., 2025), *Streptomyces microflavus* CLSD-1 (Sihan et al., 2025; Yuxin et al., 2025), and *Aspergillus niger* H8 (Ting et al., 2025), while *Bacillus cereus* S1 exhibited significant lignin and cellulose degradation capabilities (Xin et al., 2025).

Within the tobacco industry, the degradation of tobacco stem cellulose is imperative for the valorization of waste. Research has documented the utilization of exogenous microbial consortia or particular strains for the degradation of tobacco stems (Chunli et al., 2019; Xiaoxiao et al., 2025). For example, the thermotolerant *Bacillus velezensis* HF-09 has been studied for this purpose (Changwei et al., 2021). However, studies investigating indigenous cellulolytic bacteria isolated from tobacco itself remain scarce. The utilization of native tobacco-associated bacteria may confer certain benefits with regard to fermentation safety and adaptation to the tobacco substrate, potentially reducing ecological risks in comparison with the introduction of exogenous microbes. At this time, a bacterium capable of producing multiple enzymes is needed, because a single bacterium is not only an ‘enzyme producer ‘, but also a highly integrated and adaptive ‘micro-bioprocessing plant ‘tailored to deal with complex biomass. This makes it have a clearer and more direct application prospect and commercial value in the field of industrial biorefinery and waste recycling than a single enzyme preparation or microorganism (or microbial flora) that requires artificial compounding.

The objective of this study was to isolate and identify a potent cellulase-producing strain directly from tobacco leaves. The isolate was characterized through a range of analytical methods, including morphological, physiological, and biochemical assays, in addition to 16S rRNA gene sequencing and phylogenetic analysis. Whole-genome sequencing and bioinformatic analysis were conducted to elucidate the genetic basis of its cellulolytic capability. This comprehensive approach provides fundamental insights into the strain’s cellulase system and supports its potential application in the biorefinery of tobacco waste.

## 2 Materials and methods

### 2.1 Experimental materials and reagents

Fresh leaves of the C3F grade (middle orange, grade 3) were collected from tobacco (variety: Yunyan 87), which was sourced from the Yunnan production area in China.

The following media were utilized in the present study: Nutrient Broth (B) Medium contained 10 g of peptone, 3 g of beef extract, and 5 g of NaCl per liter of distilled water, with a pH of neutral. Nutrient Agar (NA) was prepared by adding 15 g agar to 1 L of Nutrient Broth (B) Medium prior to sterilization. The Carboxymethyl Cellulose (CMC) Selective Medium was as follows: 10.0 g sodium carboxymethyl cellulose (CMC-Na), 1.0 g K_2_HPO_4_, 1.0 g NH_4_NO_3_, 0.2 g MgSO_4_·7H_2_O, 0.02 g CaCl_2_, and 15.0 g agar per liter of distilled water (pH neutral). The medium was sterilized by autoclaving at 121 °C for 30 min. The above reagents were purchased from China Pharmaceutical Group Chemical Reagents Co., Ltd.

### 2.2 Strain screening and identification

#### 2.2.1 Strain screening

The strain screening was performed in accordance with a method described by Peng et al. with modifications (Yongyan et al., 2023). In summary, 10 g of tobacco leaves were aseptically added to a conical flask containing 90 mL of sterile water and incubated at 30 °C with 180 rpm for 24 h. Thereafter, 10 mL of the culture was serially diluted to concentrations of 10^−4^, 10^−5^, and 10^−6^. Aliquots (100 μL) from each dilution were spread onto CMC selective medium plates (triplicate per dilution) and incubated upside down at 30 °C for 48–72 h. Distinct single colonies were selected and repeatedly streaked on NA plates for purification.

The purified isolates were spot-inoculated onto fresh CMC plates, followed by incubation at 30 °C for a period of 4 days. The preliminary assessment of the cellulolytic activity was conducted by measuring the diameter of the hydrolysis zone (D) and the colony diameter (d). Isolates exhibiting elevated D/d ratios were selected for subsequent analysis. The preparation of seed cultures involved the inoculation of a single colony into NB, followed by incubation at 30 °C with 180 rpm agitation for a duration of 12 h. Thereafter, the seed culture was then transferred (0.3 mL inoculation volume) into 30 mL fresh NB and fermented for a period of 24 h, employing the same conditions as previously outlined. The fermentation broth was centrifuged at 4 °C and 4,000 rpm for 20 min to collect the cell pellet. The resulting suspension was centrifuged at 4 °C and 8,000 rpm for 10 min, and the supernatant was collected as the crude enzyme extract. Carboxymethylcellulase (CMCase) was quantified following the DNS method according to Miller et al. (1960) and to the International Union of Pure and Applied Chemistry with certain modification. Briefly, the main objective of this method is to estimates extent saccharification by measuring the total amount of reducing sugars obtained from the hydrolysis. For cellulase activity determination, each test tube contained 300 μL of 1% (w/v) sodium carboxymethyl cellulose solution (pH = 5.0) and 100 μL of enzyme solution. For pectinase activity determination, the substrate was replaced with 300 μL of 1% (w/v) pectin solution, while the enzyme solution volume (100 μL) remained unchanged. Both mixtures were incubated at 50 °C for 30 min to initiate the reaction. After reaction, 400 μL of 3,5-dinitrosalicylic acid (DNS) solution was added to each tube; the mixtures were heated in a boiling water bath at 100 °C for 5 min and cooled with running water. The solutions were diluted to a final volume of 10 mL, and the absorbance was measured at 540 nm to determine activities, respectively (Yubo and Xiuyan, 2008).

#### 2.2.2 Strain identification

Morphological and physiological biochemical characterization: The isolate was cultivated on NA at 30 °C for 24 h. Morphological observation was conducted using scanning electron microscopy (SEM), as previously described by Zhao et al. (2024). Physiological and biochemical tests were then performed, including glucose fermentation, catalase, oxidase, Voges-Proskauer (V-P), methyl red, starch hydrolysis, gelatin liquefaction, nitrate reduction, citrate utilization, urease, indole production, and Gram staining. These tests were performed according to standard procedures outlined in Bergey’s Manual of Determinative Bacteriology (Buchanan, 1984) and Manual for Systematic Identification of Common Bacteria (Xiuzhu and Miaoying, 2001).

16S rDNA Gene Sequencing and Phylogenetic Analysis: Genomic DNA was extracted by subjecting 10 μL of a 24-h NA broth culture to heating at 95 °C for 7 min, followed by centrifugation at 10,000 rpm for 10 min. The resultant pellet was then utilized as the DNA template. The 16S rDNA was amplified via PCR using universal primers 27F (5’-AGAGTTTGATCMTGGCTCAG-3′) and 1492R (5’-GGTTACCTTGTTACGACTT-3′). The PCR mixture (50 μL) comprised 25 μL of 2 × Taq Master Mix, 2 μL of each primer (10 μmol/L), 2 μL of DNA template, and 19 μL of ddH_2_O, The kit used in the experiment is Taq PCR Master Mix Kit (Qiagen, Germany). The amplification program comprised an initial denaturation at 94 °C for 3 min, followed by 30 cycles of denaturation at 94 °C for 30 s, annealing at 55 °C for 30 s, and extension at 72 °C for 1 min. The program concluded with a final extension at 72 °C for 5 min. The subsequent analysis of the PCR products was conducted by Sangon Biotech (Shanghai) Co., Ltd. The resulting sequence was then compared with those in the NCBI database using BLAST. The phylogenetic tree of the strain was constructed using 31 housekeeping genes.

### 2.3 Growth curve determination

A single colony of the strain was inoculated into 30 mL NB and incubated at 30 °C, 180 rpm for 12 h to prepare the seed culture. The seed culture was then transferred (0.3 mL inoculation, OD_600_ = 0.01) into fresh NB. During the incubation at 30 °C and 180 rpm, samples were collected at 0, 2, 4, 6, 8, 10, 12, 14, 16, 18, 20, 22, 24, 26, 28, 30, 32, 34, 36, 38, 40, 42, 44, 46, 48, and 50 h. The optical density at 600 nm (OD_600_) of each sample was measured using a UV spectrophotometer to create a growth curve. The specific growth rate (*μ*) and the generation time (g) of the strain in the logarithmic growth phase were calculated according to the method of Zhong et al. (2011).

### 2.4 Determination of enzyme activities

The selected strain was inoculated from an NA plate into 30 mL of NA broth and cultured at 30 °C, 180 rpm for 24 h to prepare the seed culture. The seed culture was then transferred (at a rate of 2% inoculation, OD600 = 0.02) into fresh Nutrient Broth (B), whereupon it was fermented under the same conditions for 24 h. The culture was then centrifuged at 8,000 rpm and 4 °C for 10 min to obtain the crude enzyme. The activities of cellulase was assayed according to the method described by Miller et al. (1960). The determination of protease activity was conducted according to the stipulated protocols outlined in the Chinese National Standard GB/T 23527-2009. The activities of cellulase, amylase, pectinase, and xylanase were measured using the DNS method (Dongdong et al., 2024; Yubo and Xiuyan, 2008; Zhaowei et al., 2013; Lei et al., 2022). The activities of peroxidase and manganese peroxidase were assayed according to the method described by Jing et al. (2021), while laccase activity was determined as referenced by Xiaofeng et al. (2019). The activity of tannase was determined according to the method of Mahmoud et al. (2023). The activity of nicotine dehydrogenase was determined according to the method of Ke et al. (2023).

### 2.5 Whole-genome analysis

#### 2.5.1 DNA extraction

Bacterial cells from a 50 mL log-phase culture were harvested by centrifugation at 8,000 rpm for 10 min at 5 °C. The pellet was flash-frozen in liquid nitrogen. DNA was extracted by Fast DNA® SPIN extraction kits (MP Biomedicals, Santa Ana, CA, USA) according to the manufacturer’s instructions. The purity and integrity of the DNA were checked by agarose gel electrophoresis. Qualified DNA samples were dispatched to Shanghai Majorbio Bio-pharm Technology Co., Ltd. for whole-genome sequencing on the Illumina NovaSeq 6,000 platform.

#### 2.5.2 Genome sequencing, assembly, annotation and prediction

A complete genome sequence was obtained using a combination of Illumina (second-generation) and PacBio Sequel IIe (third-generation) sequencing technologies, providing at least 100 × coverage for each platform. The quality control of the raw data was performed using the Fastp software. The *de novo* assembly was performed using SOAPdenovo, and gaps in the assembly were filled using GapCloser. The completeness of the genome assembly was assessed using the BUSCO 5.4.5 software^1^ and the data set used is Bacteria odb10. A circular genome map was generated using CGView.

Protein-coding genes were predicted and functionally annotated against the KEGG Database 20230830^2^ and the CAZy Database v12^3^ databases (Kexin et al., 2022).

### 2.6 Fermentation of tobacco stems with crude enzyme

The crude enzyme extract was prepared as described in section 2.4. Tobacco stems were evenly sprayed with the crude enzyme solution at a dosage equivalent to 25% of the stem weight (using a volume of enzyme solution constituting 20% of the final crude enzyme preparation). The stems were fermented at 45 °C for 10 h, and then dried in an oven at 80 °C for 10 min. The dried stems were equilibrated in a constant temperature and humidity chamber for 24 h. The cellulose content in the fermented stems was determined using a commercial assay kit (China Shanghai Enzyme-linked Biotechnology Co., Ltd.).

### 2.7 Statistical analysis

The data were analyzed by Microsoft Excel 2013 and R packages 1.6.2 (Zhongxu et al., 2025). Independent-samples *t-*test was performed to analyze significant differences. *p*-values were two-sided. A value of * *p* < 0.05 was considered significant. Data were expressed as mean ±  standard deviation. Microsoft Excel 2013 was used for plotting (Xinyu et al., 2024).

## 3 Results

### 3.1 Screening and selection of cellulase-producing strain

**Figure 1:**
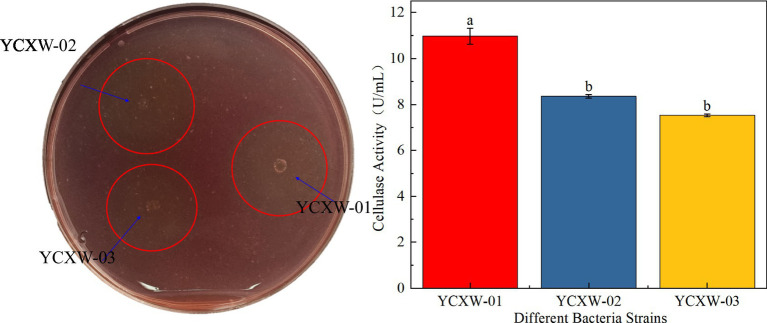
Analysis of cellulase production by strains: **(A)** CMC plates; **(B)** cellulase activity. Different lowercase letters indicated that there were significant differences between treatments at the *p* < 0.05 level (Tukey HSD test).

Initially, three cellulase-producing strains were isolated from the tobacco leaf samples. Among them, strain YCXW-01 exhibited the highest hydrolytic capacity on CMC plates, as indicated by the largest D/d of 2.25 (Table 1 and Figure 1A). In accordance with the findings of the preliminary screening, YCXW-01 also demonstrated the highest cellulase activity (10.97 U/mL) in quantitative assays, Followed by YCXW-02 (8.36 U/mL) and YCXW-03 (7.53 U/mL) (Figure 1B). Consequently, strain YCXW-01 was selected for all subsequent investigations.

**Table 1:** Screening of cellulose-degrading strains.

Strain	Colony Diameter d (mm)	Hydrolysis Zone Diameter D (mm)	D/d
YCXW-01	7.03 ± 0.19	15.85 ± 0.46	2.25
YCXW-02	6.88 ± 0.06	14.26 ± 0.29	2.07
YCXW-03	4.08 ± 0.08	7.35 ± 0.23	1.8

### 3.2 Identification of strain YCXW-01

#### 3.2.1 Morphological characteristics

**Figure 2:**
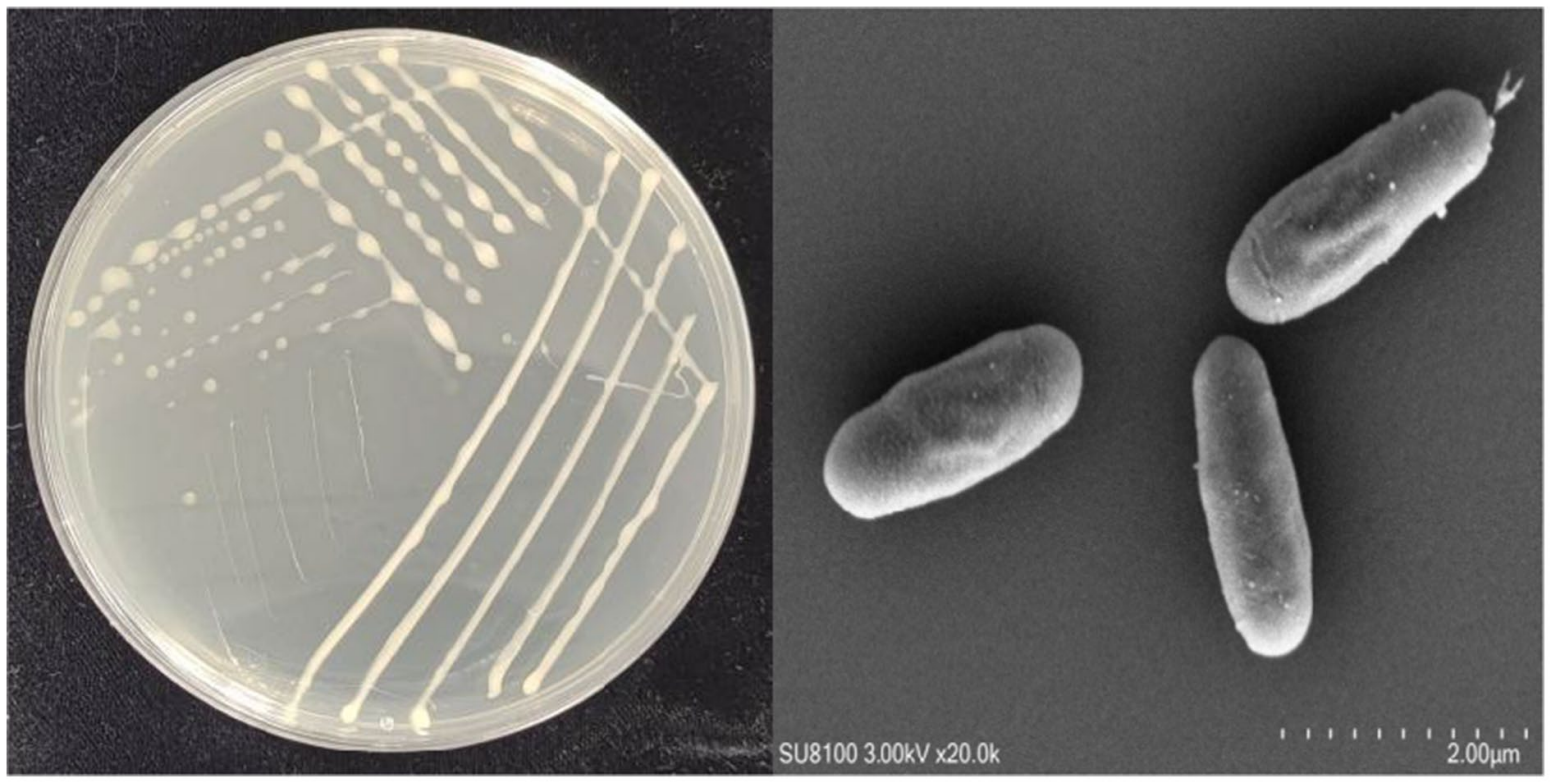
Morphological characteristics of strain YCXW-01: **(A)** streak plate culture, **(B)** scanning electron microscope.

On NA medium, colonies of YCXW-01 manifested a circular morphology, with a creamy-white hue, convex form, and opaque characteristics. The surface of these colonies was characterized by a smooth and moist texture (Figure 2A). The SEM revealed that the cells were rod-shaped with rounded ends and a smooth surface, displaying fine textures or uneven structures on the cell wall, characteristics typical of the genus *Bacillus* (Figure 2B).

#### 3.2.2 Physiological and biochemical characteristics

The physiological and biochemical profile of YCXW-01 is summarized in Table 2. The strain was identified as Gram-positive. The analysis yielded positive results for glucose fermentation, starch hydrolysis, catalase, Voges-Proskauer (V-P) test, gelatin liquefaction, citrate utilization, and nitrate reduction. Conversely, the experiment yielded negative results for the methyl red test, oxidase, urease, and indole production. This pattern of characteristics is consistent with those of *Bacillus subtilis* (Guangwei et al., 2024; Junfang et al., 2020).

**Table 2:** Physiological and biochemical characteristics of strain YCXW-01.

Test	Result	Test	Result
Glucose fermentation	+	Oxidase	−
Gram staining	+	V-P test	+
Catalase	+	Indole production	−
Gelatin liquefaction	+	Nitrate reduction	+
Citrate utilization	+	Urease	−
Starch hydrolysis	+	Methyl red test	−

#### 3.2.3 16S rRNA gene sequencing and phylogenetic analysis

**Figure 3:**
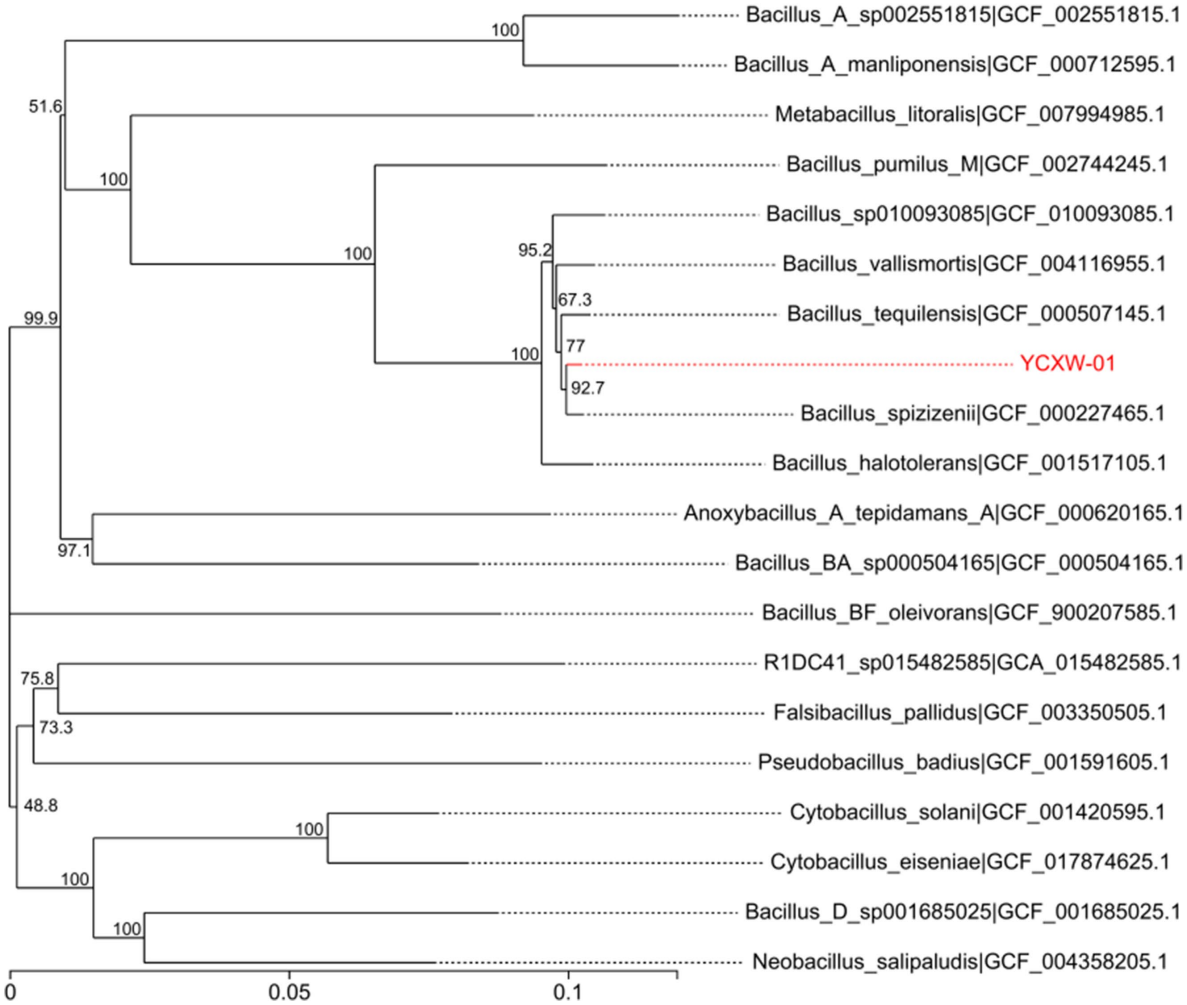
Phylogenetic tree based on 31 house-keeping genes (dnaG, frr, infC, nusA, pgk, pyrG, rplA, rplB, rplC, rplD, rplE, rplF, rplK, rplL, rplM, rplN, rplP, rplS, rplT, rpmA, rpoB, rpsB, rpsC, rpsE, rpsI, rpsJ, rpsK, rpsM, rpsS, smpB, tsf). The position of strain YCXW-01 is shown with respect to other closely related species. The tree was generated using the neighbor-joining method. Numbers at the nodes indicated bootstrap values, expressed as percentages of 1,000 replications. Bar, 0.05 changes per nucleotide position.

The 16S rRNA gene sequence of YCXW-01 was deposited in the SRA database under accession number SUB15310007. The results of the BLAST analysis and the construction of the phylogenetic tree demonstrated that the strain shared 99% sequence similarity with *Bacillus spizizenii* (GCF_000227465.1), a member of the *Bacillus subtilis* species complex (Figure 3). In consideration of the morphological and physiological data, strain YCXW-01 was unequivocally identified as *Bacillus subtilis*.

### 3.3 Growth curve of strain YCXW-01

**Figure 4:**
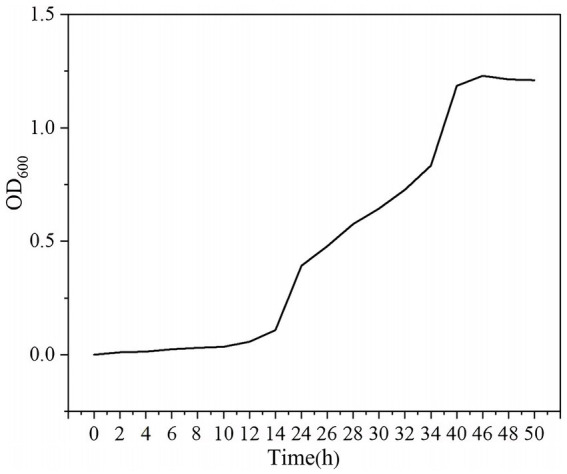
The growth curve of strain YCXW-01 in NB at 30 °C and 180 rpm for 0–50 h.

The growth dynamics of YCXW-01 in NB are demonstrated in Figure 4. The strain exhibited a lag phase of approximately 12 h, during which it adapted to the new environment. This was followed by a rapid exponential growth phase from 12 to 46 h, during which vigorous metabolism and cell division occurred. The culture attained its maximum OD_600_ at 46 h, entering the stationary phase, likely as a consequence of nutrient depletion and the accumulation of metabolic by-products. By calculation, the specific growth rate of the strain in the logarithmic phase was 0.067 h^−1^, and the generation time was 10.34 h.

### 3.4 Extracellular enzyme production profile

**Figure 5:**
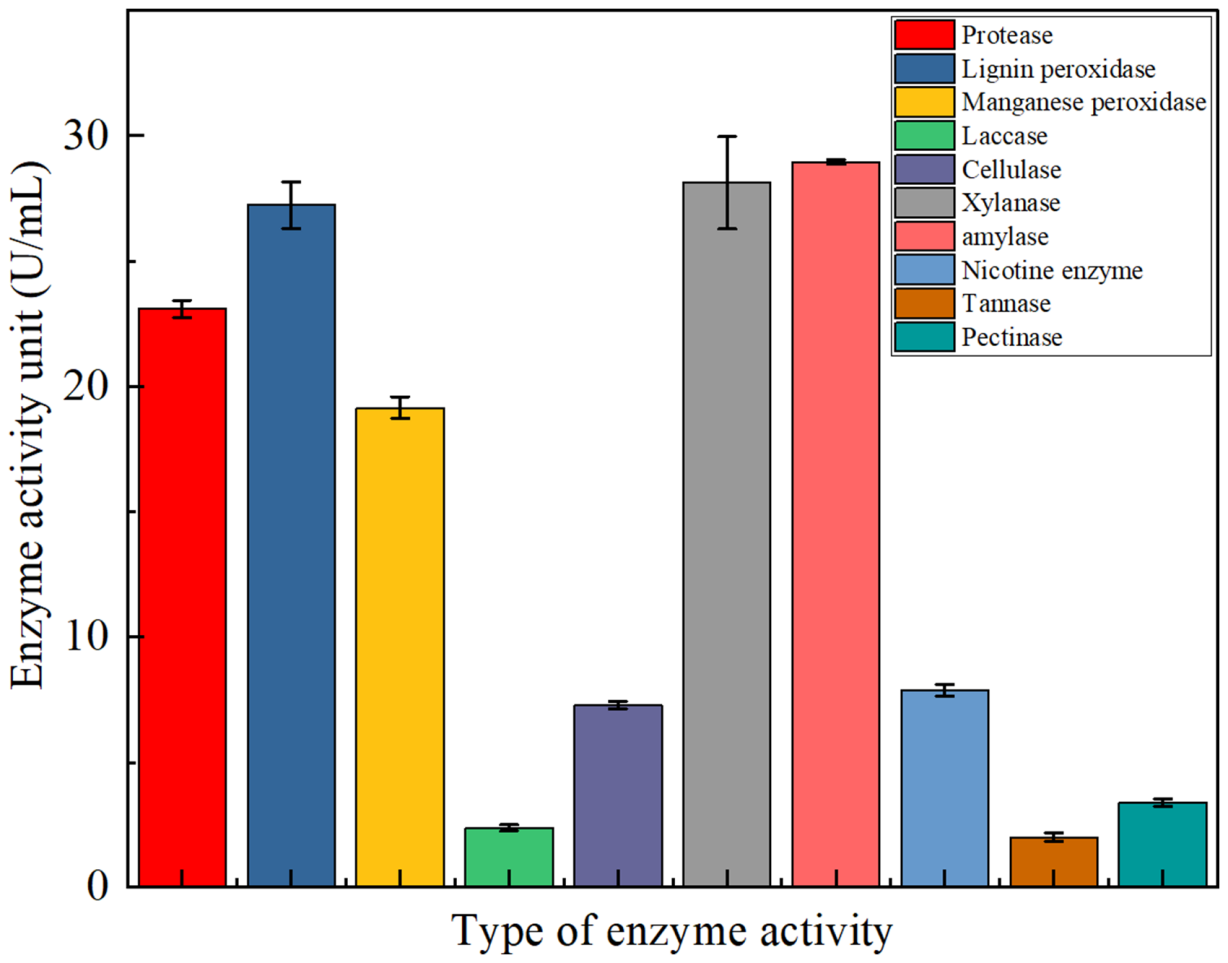
The type of extracellular enzyme activity produced by strain YCXW-01 cultured in NA medium at 30 °C and 180 rpm for 24 h, The enzyme activity was measured three times each time, the statistical method used is ANOVA.

The potential of YCXW-01 to produce a wide range of extracellular enzymes was evaluated (Figure 5). In addition to cellulase, the strain exhibited significant production of protease (23.11 U/mL), amylase (28.96 U/mL), xylanase (23.68 U/mL), and lignin peroxidase (27.24 U/mL). Furthermore, the study revealed the production of pectinase, manganese peroxidase, and laccase, albeit at lower levels of activity. This multi-enzyme profile suggests a strong potential for synergistically degrading complex plant cell wall components like cellulose, hemicellulose, lignin, and pectin in tobacco stems.

### 3.5 General genomic features

**Figure 6:**
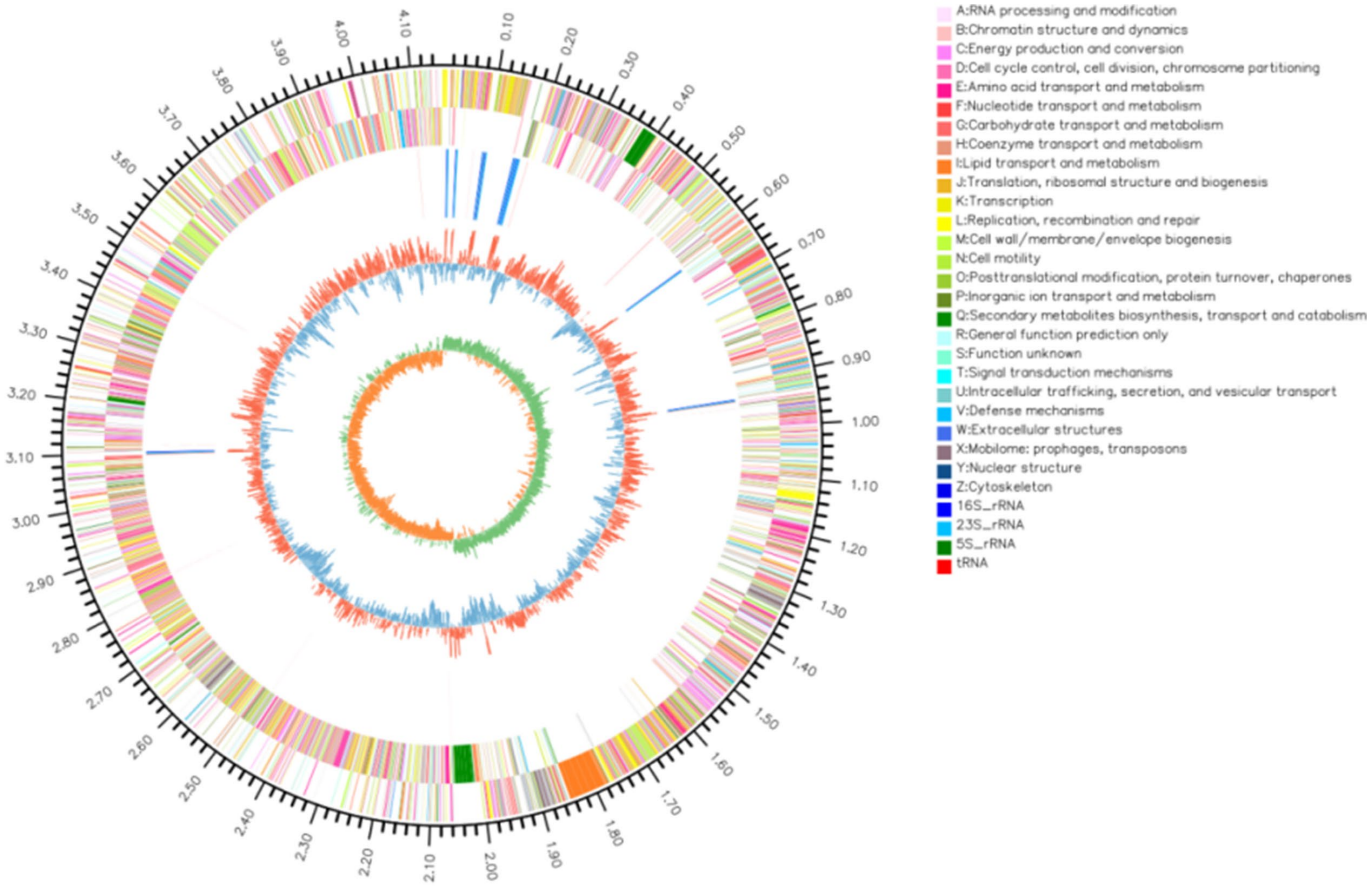
The circular map of strain YCXW-01 completely showed the genomic characteristics. From outside to inside, they were: chromosome region (outermost circle), positive-strand protein-coding gene (second circle), negative-strand coding region (third circle), non-coding RNA (including rRNA and tRNA) (fourth circle), GC content (fifth circle) and GC offset value (sixth circle). The circles of different colors correspond to the coding area categories in the second and third circles, respectively. The color and height of the fifth peak represent the comparison of GC content with the average (red: higher, blue: lower), and the difference value.

The complete genome of strain YCXW-01 was sequenced using a combination of Illumina and PacBio approaches. The genome is constituted of a single circular chromosome measuring 4,156,553 base pairs (bp) with a guanine-cytosine (GC) content of 44.46% (Figure 6). A total of 4,114 protein-coding genes were predicted, covering 88.19% of the genome. The mean gene length was found to be 891 bp. Furthermore, the genome contains 207 non-coding RNAs, including 85 tRNAs, 30 rRNAs (which are organized in 10 operons), and 92 sRNAs.

### 3.6 KEGG functional annotation

**Figure 7:**
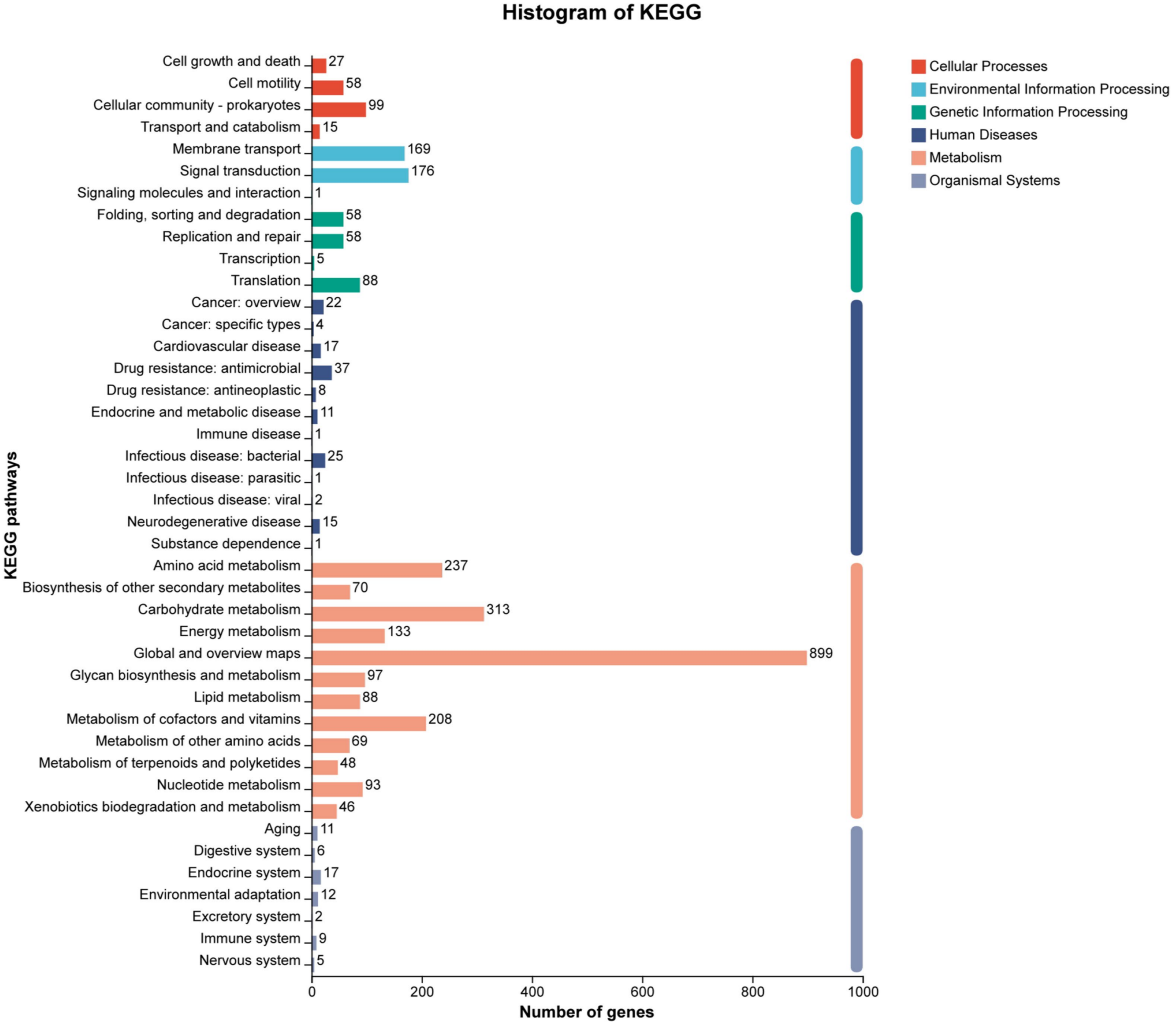
KEGG cluster annotation of strain YCXW-01. The horizontal axis represents the secondary classification of the KEGG pathway, and the vertical axis represents the number of genes labeled under this classification. Columns of different colors correspond to the first-level classification of the KEGG pathway, and the rightmost column shows the number of genes in different levels of classification. Since the same gene may be labeled to multiple secondary classifications, de-redundancy processing is performed when calculating the number of genes in the primary classification.

The KEGG pathway annotation assigned 3,018 genes to six major functional categories (Figure 7). The category of ‘Metabolism’ was the most prominent category, containing 2,301 genes. Sub-categories within metabolism with high gene counts included ‘Carbohydrate metabolism’ (313 genes) and ‘Amino acid metabolism’ (237 genes). Notably, 148 genes were found to be enriched in four pathways directly related to cellulose and sugar metabolism: starch and sucrose metabolism (ko00500, 49 genes), citrate cycle (TCA cycle) (ko00020, 24 genes), glycolysis/gluconeogenesis (ko00010, 43 genes), and pentose phosphate pathway (ko00030, 32 genes) (Supplementary Table S1).

### 3.7 CAZyme annotation and cellulase-related genes

**Figure 8:**
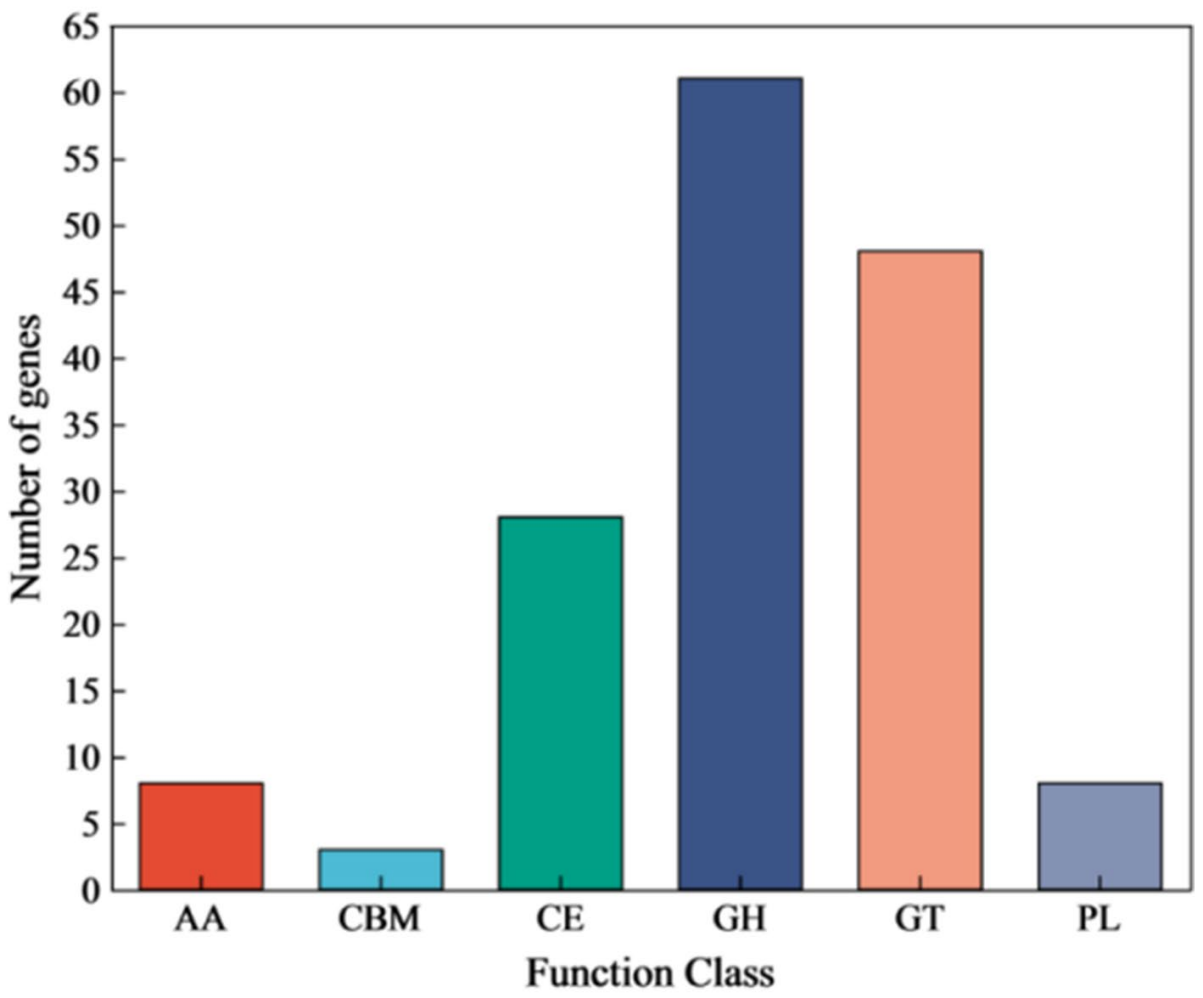
Genome CAZy annotation classification of strain YCXW-01. AA, enzymes with auxiliary activity; CBM, carbohydrate-binding module; CE, carbohydrate esterase; GH, Glycoside Hydrolases; GT, glycosyl transferase; PL, polysaccharide lyase.

Annotation against the CAZy database identified 148 genes encoding Carbohydrate-Active Enzymes (CAZymes) (Figure 8). These included 61 Glycoside Hydrolases (GHs), 48 Glycosyl Transferases (GTs), and 28 Carbohydrate Esterases (CEs). A detailed analysis was conducted to identify the key genes potentially involved in cellulose and hemicellulose degradation (Table 3). These genes encode enzymes such as beta-glucosidase (GH1), endoglucanase (GH5), endo-1,4-beta-xylanase (GH11), xylan 1,4-beta-xylosidase (GH43), and acetyl xylan esterase (CE7). The GH1 and GH43 families were found to contain the highest number of genes (4 each), thus suggesting their potential pivotal role in the strain’s lignocellulolytic system.

**Table 3:** Analysis of genes related to cellulase production of strain YCXW-01.

Gene ID	Gene name	Enzyme definition	Family
gene0382	bglA	Beta-glucosidase (EC 3.2.1.21)	GH1
gene0629	bglA	Beta-glucosidase (EC 3.2.1.21)	GH1
gene2011	–	Endoglucanase (EC:3.2.1.4)	GH5
gene3971	celF	6-phospho-beta-glucosidase (EC:3.2.1.86)	GH4
gene4046	bglA	Beta-glucosidase (EC 3.2.1.21)	GH1
gene4132	bglA	Beta-glucosidase (EC 3.2.1.21)	GH1
gene1949	xynB	Xylan 1,4-beta-xylosidase (EC:3.2.1.37)	GH43
gene2013	xynC	Glucuronoarabinoxylan endo-1,4-beta-xylanase (EC:3.2.1.136)	GH30
gene2014	xynD	Arabinoxylan arabinofuranohydrolase (EC:3.2.1.55)	GH43
gene2088	xynA	Endo-beta-1,4-xylanase (EC 3.2.1.8)	GH11
gene2951	abnA	Arabinan endo-1,5-alpha-L-arabinosidase (EC:3.2.1.99)	GH43
gene4058	abnA	Arabinan endo-1,5-alpha-L-arabinosidase (EC:3.2.1.99)	GH43
gene0357	cah	Acetyl xylan esterase (EC 3.1.1.72)	CE7

### 3.8 Degradation of tobacco stems by crude enzyme

**Figure 9:**
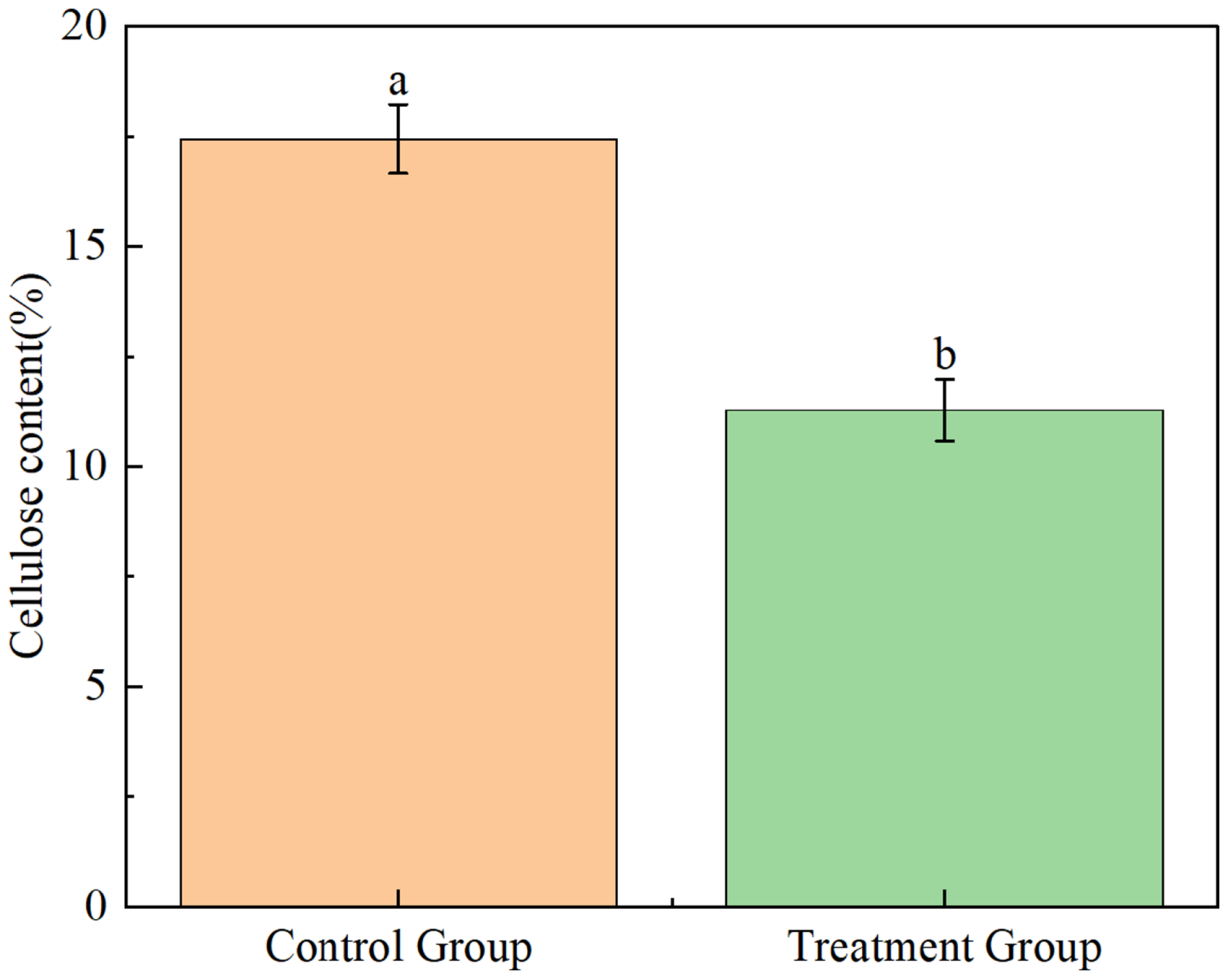
The extracellular crude enzyme solution of strain YCXW-01 actually analyzed the cellulose degradation ability in tobacco stem (the control group was kept under the same conditions without applying the enzyme). Different lowercase letters indicated that there were significant differences between treatments at the p < 0.05 level (Tukey HSD test).

The practical application potential of YCXW-01 was evaluated by treating tobacco stems with its crude extracellular enzyme extract. The treatment resulted in a significant 35.3% reduction in cellulose content compared to the untreated control (Figure 9), unequivocally demonstrating the high efficiency of this strain in degrading cellulose within a complex natural substrate like tobacco stems.

## 4 Discussion

In this study, a highly efficient cellulose-degrading bacterium, *Bacillus subtilis* YCXW-01, was successfully isolated and identified from the surface of tobacco leaves. The strain not only exhibited cellulase activity of up to 10.97 U/mL, but also had the ability to secrete various hydrolytic enzymes such as protease, amylase and xylanase, forming a powerful multi-enzyme degradation system.

The application of its crude enzyme solution to tobacco straw can significantly reduce the cellulose content by 35.3%, which fully proves its great application potential in the bioconversion of tobacco waste. The excellent degradation efficiency of YCXW-01 is superior to many reported strains. For example, its degradation rate is much higher than that of *Bacillus velezensis* CL-4 with a degradation rate of 21.33%, in a longer period of 48 h (Long et al., 2022). The multi-enzyme system of *B. subtilis* YCXW-01 provides an attractive solution for its application in industrial-scale biological processes and waste treatment. Its core value lies in the ability to produce a versatile enzyme kit that can synergistically attack multiple biomass components through a single, safe, and efficient fermentation process. This has laid a solid foundation of microbial resources for the development of more economical and environmentally friendly waste recycling technology and the realization of ‘turning waste into treasure’ of agricultural and food industry by-products.

### 4.1 Genome-wide analysis

The Genome-wide analysis reveals the mechanism of its efficient degradation at the molecular level. We identified several key CAZyme genes belonging to GH1, GH5 and GH43 families in YCXW-01. Among them, the presence of multiple GH1 family *β*-glucosidase genes is particularly critical, because the enzyme can relieve the feedback inhibition of cellobiose on the cellulase system, which is often the rate-limiting step for efficient cellulose saccharification (Yu-Kyoung et al., 2012). In addition, the discovery of endo-xylanase (GH11) and debranching enzyme (GH43, CE7) genes explained the ability of the strain to attack hemicellulose, which helped it to more effectively expose and degrade internal cellulose microfibrils. Its core advantage lies in the complex enzyme system encoded by the genome that can work synergistically. Compared with *M. luteus* FJAT-25102 and other strains that are only good at cellulase gene diversity (Jianmei et al., 2025), the genome of YCXW-01 contains key hemicellulase genes such as protease, amylase and xylanase (GH11, GH43). This multi-enzyme synergy is essential for breaking the complex network of cellulose, hemicellulose, lignin and pectin in tobacco cell wall (Yi et al., 2008). It is worth noting that YCXW-01, as *B. subtilis*, has natural strong secretory capacity and is easy to form stress-resistant spores (Zeng et al., 2024; Liwen et al., 2025), making it very suitable for industrial-scale fermentation and environmental applications. Based on the complete genome data provided in this study, the mature *Bacillus subtilis* gene manipulation tool (Gongwei et al., 2025) can be used in the future to further enhance the cellulose degradation efficiency of YCXW-01 by overexpressing the above key GH1 and GH43 genes, so as to provide a better microbial solution for the high-value utilization of tobacco straw. Compared with *B. subtilis* CRN1 and *B. subtilis* CRN7 reported by Shweta Srivastava et al., YCXW-01 has a richer GHs gene pool, especially multiple GH1 (*β*-glucosidase) genes, which can effectively relieve the feedback inhibition of cellobiose and is the key to efficient and thorough saccharification of cellulose. Its comprehensive gene spectrum (GH5, GH11, GH43, CE7) provides the genetic basis for its excellent multi-enzyme activity (Shweta et al., 2022).

Different from other *B. subtilis* cellulose-decomposing bacteria (Zeng et al., 2024), *B. subtilis* YCXW-01 is not an ordinary cellulose-decomposing bacteria, but a ‘specialized’ strain with a strong and balanced multi-enzyme system, a genetic blueprint for efficient degradation, and excellent degradation performance for tobacco waste in practical applications. Its ‘totipotent ‘zymography, key genetic characteristics (especially multi-copy GH genes), and efficient degradation of native substrates together constitute its core advantages, making it have outstanding application potential in agricultural waste biorefinery, especially in tobacco stem treatment.

Genomic information explains why it has such a strong potential, and enzyme activity data and application experiments prove that it is indeed so powerful and quantify its power. This together constitutes a solid scientific foundation to support its application value.

## 5 Conclusion

In conclusion, the results of this study showed that a highly efficient cellulose-degrading strain, *Bacillus subtilis* YCXW-01, was successfully isolated and identified from the surface of tobacco leaves. The strain not only showed high cellulase activity (10.97 U/mL), but also had the ability to produce various extracellular enzymes such as protease, amylase and xylanase, showing its comprehensive potential in degrading complex plant cell wall components. Through genome-wide analysis, researchers found that its genome contains multiple glycoside hydrolase genes related to cellulose and hemicellulose degradation, especially GH1 and GH43 family genes may play a key role in cellulose degradation. In practical application, the crude enzyme solution of the strain was used to treat tobacco straw, which significantly reduced the cellulose content by 35.3%, which verified its high efficiency and application prospect in the resource utilization of tobacco waste. Therefore, *B. subtilis* YCXW-01 not only provides excellent microbial resources for the biodegradation of tobacco straw, but also lays a theoretical foundation for further improving its degradation ability by genetic engineering.

## Data Availability

The datasets presented in this study can be found in online repositories. The names of the repository/repositories and accession number(s) can be found in the article/Supplementary material.

## Footnotes

1
https://busco.ezlab.org/

2
https://www.kegg.jp/

3
http://www.cazy.org/
