# Supplementary material for: A multi-enzyme producing Bacillus subtilis YCXW-01: Isolation, genomic characterization, and potentials in tobacco stem degradation
Source: Front Microbiol. 2025 Dec 3;16:1717865. doi: 10.3389/fmicb.2025.1717865 (PMC12709117; doi:10.3389/fmicb.2025.1717865)
Supplement: Supplementary file 1 [file Table_1.pdf]

Table S1 Genes related to the pathways of cellulose metabolism

Coad	KEGG ID	Category	ID
1	Ko00500	Starch and sucrose metabolism	gene0210, gene3917,gene3953…
2	Ko00020	Citrate cycle (TCA cycle)	gene0869、gene1562 和 gene3370…
3	Ko00010	Glycolysis / Gluconeogenesis	gene0344、gene0382 和 gene0629…
4	Ko00030	Pentose phosphate pathway	gene0061、gene0322 和 gene0436…
